# Automatic polyp image segmentation and cancer prediction based on deep learning

**DOI:** 10.3389/fonc.2022.1087438

**Published:** 2023-01-12

**Authors:** Tongping Shen, Xueguang Li

**Affiliations:** ^1^ School of Information Engineering, Anhui University of Chinese Medicine, Hefei, China; ^2^ Graduate School, Angeles University Foundation, Angeles, Philippines; ^3^ School of Computer Science and Technology, Henan Institute of Technology, Xinxiang, China

**Keywords:** colon polyps, attention mechanism, HarDNet, image segmentation, deep learning

## Abstract

The similar shape and texture of colonic polyps and normal mucosal tissues lead to low accuracy of medical image segmentation algorithms. To solve these problems, we proposed a polyp image segmentation algorithm based on deep learning technology, which combines a HarDNet module, attention module, and multi-scale coding module with the U-Net network as the basic framework, including two stages of coding and decoding. In the encoder stage, HarDNet68 is used as the main backbone network to extract features using four null space convolutional pooling pyramids while improving the inference speed and computational efficiency; the attention mechanism module is added to the encoding and decoding network; then the model can learn the global and local feature information of the polyp image, thus having the ability to process information in both spatial and channel dimensions, to solve the problem of information loss in the encoding stage of the network and improving the performance of the segmentation network. Through comparative analysis with other algorithms, we can find that the network of this paper has a certain degree of improvement in segmentation accuracy and operation speed, which can effectively assist physicians in removing abnormal colorectal tissues and thus reduce the probability of polyp cancer, and improve the survival rate and quality of life of patients. Also, it has good generalization ability, which can provide technical support and prevention for colon cancer.

## 1 Introduction

Colon cancer is a malignant tumor on the colonic mucosa, mostly formed by adenomatous polyps, characterized by high incidence and high lethality, and is currently one of the three most prevalent malignancies in the world (1). Colon polyps are convexities that grow in the colonic mucosa, and the gold standard for early screening of colorectal cancer is the use of colonoscopy to detect polyps larger than 5 mm in diameter in the intestine (2). There is a link between the accurate detection rate of polyps and the incidence of colon cancer. Dougla et al. (3) showed that for every 1% increase in polyp detection, the prevalence of colorectal cancer would decrease by 3%. Prevention and diagnosis of colorectal cancer through early screening are very important and can improve patient survival rates. Polyps can usually be effectively detected by colonoscopic screening, but even if the polyps are of the same type, it varies in size, color, and texture. Secondly, in colonoscopic images of intestinal polyps, the contrast between the polyps and the surrounding mucosa is not strong enough and the border is blurred and not clear enough due to the intestinal mucus and the reflection of the intestinal polyps under colonoscopy. Therefore, it may cause the physician to miss the polyps and segment them inaccurately. Therefore, how to segment colon polyps quickly and accurately is important for the early prevention of colorectal cancer coming (4).

For the segmentation of colon polyp images, a large number of methods exist at home and abroad. They can be mainly included two types: early traditional algorithms and deep learning-based algorithms. The traditional segmentation methods mainly extract features such as color, shape, and texture, and then use classifiers to distinguish polyps from their surrounding non-polyp regions.

Saul et al. in 2009 proposed the use of similarity measures to discriminate colon polyps as a way to reduce the workload of physicians (5). Bashar et al. in 2010 proposed the segmentation of the capsule endoscopic images from the possible presence of polyps based on the energy distribution of the images (6). Segui et al. in 2012 used the texture and color information as the basis for screening (7). Turcza et al. in 2013 proposed to use an entropy encoder to initially screen capsule endoscopic images and filter out some highly similar images (8). Hassan et al. in 2015 proposed to perform gray value statistics on capsule endoscopic images and observe their spectral features to screen out images of polyps (9). In 2016, Qiao et al. attempted to convert capsule endoscopy images from red, green, and blue (RGB) color gamut to columnar color gamut, which enhanced the contrast between the polyp part and normal bowel part (10). In 2014, Mamonov et al. designed a binary classifier to label each frame of a colonoscopy video based on the geometric analysis and texture content on each frame as containing or not containing polyps (11). In 2015, Tajbakhsh et al. used an automatic method to detect colonoscopy video polyps based on shape and contextual information (12). In 2015, Bernal et al. designed a model based on the appearance of polyps using the median depth of the valley accumulation window. The algorithm associated with the probability of polyp presence WM-DOVA energy map to obtain the specific range of distinction between polyps and surrounding tissue and thus determine the specific location of polyps (13).

In these early algorithms for image segmentation of colon polyps, more reliance was placed on manual efforts to extract feature information from the images, followed by classifiers to segment polyps and tissues.

Although the traditional algorithms are relatively simple in implementation, they cannot combine the effective features of the polyp region by considering them simultaneously. At the same time, since the designed classifiers usually produce effects only on the specified polyp dataset, the traditional algorithms have poor generalization and the segmentation effects on other polyp datasets are significantly reduced.

Deep neural network techniques are rapidly developing and are increasingly using in polyp image segmentation.

Brandao et al. proposed Fully Convolutional Networks (FCN) for recognition and segmentation of polyp images in 2017 and achieved good polyp segmentation results (14). Wang used Dynamic Convolution Neural Network (DCNN) model in 2018 to validate and evaluate two publicly available polyp datasets (15). In 2018, Zhou et al. designed an encoder-decoder network structure UNet ++, which employs a deeply supervised training approach that allows supervised learning of the model’s multi-branch output. The model applies more jump connections between high-dimensional and low-dimensional information, reduces the feature error between semantic information, and better improves the segmentation accuracy of colon polyp images (16). In 2019, Jha et al. improved the segmentation accuracy of colon polyp images by using residual blocks, compressed excitation blocks, null spatial pyramid pooling and attention mechanism to design the ResUNet++ network, which significantly improved the segmentation of colon polyps (17). Fang et al. in 2019 designed the Selective Feature Aggregation Network (SFANet) to predict and segment regions and boundaries of polyp images (18). In 2020, to capture more effective semantic information, Jha et al. further designed the DoubleU-Net model, which effectively bridges two U-shaped structures through void space pyramidal pooling, and validated the performance of the model on a colon polyp image segmentation dataset, but the model’s parameter number was large (19).

Nadimi et al. proposed an improved AlexNet compounded with migration learning, data preprocessing, and data enhancement to detect capsule endoscopic polyps in 2020 and achieved 98.0% accuracy and 98.1% sensitivity (20). Owais et al. proposed a method in 2020 and achieved better results in 52471 endoscopic capsule images to achieve better polyp detection (21). Yanada used a novel deep learning automatic detection method in 2020 and produced a dataset to confirm the method’s effectiveness for polyp images, thus improving the early detection rate of intestinal tumors (22). Lee et al. proposed in 2021 the use of a variable depth of CNN for cancer risk level assessment of various intestinal diseases such as polyps (23). Lai et al. proposed in 2021 that the raw capsule endoscopic images were multi-channel separated and then fed into a deep CNN for training, which resulted in more accurate polyp identification (24).

In 2020, Fan designed the PraNet to improve segmentation accuracy on five colon polyp datasets, allowing real-time prediction of polyps in colonoscopy detection videos (25). In 2021, Zhao et al. designed the Multi-scale Subtraction Network (MSNet). The different levels of perceptual fields are then set in a pyramidal fashion to obtain rich multi-scale difference information (26). In 2021, Huang et al. improved on PraNet by designing a simple encoder-decoder model using Harmonic DenseNet (HarDNet) (27) HarDNet-MSEG. The original Res2Net50 (23) backbone network was replaced using the HarDNet68 backbone network, and the attention mechanism was removed to achieve more accurate polyp image segmentation. However, the method still does not work well for the diversity of polyp size, shape, and texture (28).

Instead of relying on the manual acquisition of features, deep learning algorithms use colon polyp datasets to continuously train on the established neural network model and finally optimize the model with the highest segmentation accuracy. However, many polyp segmentation networks based on deep learning algorithms focus on developing complex network structures to achieve better polyp segmentation performance, resulting in increased network model parameters and larger computation, which directly affect the computational efficiency of polyp segmentation networks. As shown in reference (29), the model parameter size of UACANet-L is 69.15M, HRNetV2-W48 model parameter size is 65.84M, while the parameter size of the proposed model in this paper is 23.11M.

To solve these problems, we propose a multi-scale coded colon polyp segmentation network combining HarDNet and attention mechanism; the segmentation network uses the U-Net network as the basic framework, including two stages of encoding and decoding. In the encoder stage, HarDNet68 is used as the backbone network to extract features using four null space convolutional pooling pyramids while improving the inference speed and computational efficiency. The attention mechanism module is added to the encoding and decoding networks so that the segmentation network can learn the global and local feature information of polyp images, thus having the information processing capability in both spatial and channel dimensions, solving the problem of encoder part of the information loss and the difficulty of small lesion segmentation.

The main contributions of this paper are as follows:

(1) Considering the impact of computation and memory access on the model design, this paper adopts HarDNet68 as the backbone network. HarDNet68 network can both learn the global feature information of polyp images and reduce the computation of the model, thus improving the operation speed of the model and the segmentation effect and accuracy of polyp images.
(2) An integrated spatial and channel attention (SCA) module is proposed, which can assign different attention weights from both spatial and channel dimensions, enabling the model to focus more on the image segmentation task. The model can be integrated into mainstream neural network segmentation tasks.
(3) The DenseASPP module is used in the segmentation network, which constitutes a dense feature pyramid, and the field obtains a larger perceptual field to improve the model’s ability to obtain information about image features.
(4) To address the problem that the targets segmented in this paper have a small proportion in the image, the combined algorithm of Dice loss and Focal loss is proposed as a loss function to reduce the weight of simple samples and improve the segmentation accuracy of small target samples.
(5) We perform analytical analysis on five polyps datasets with different sizes and performances, as well as generalization experiments, etc., to verify the effectiveness and excellence of the algorithms in this paper.

## 2 The proposed architecture

**Figure 1:**
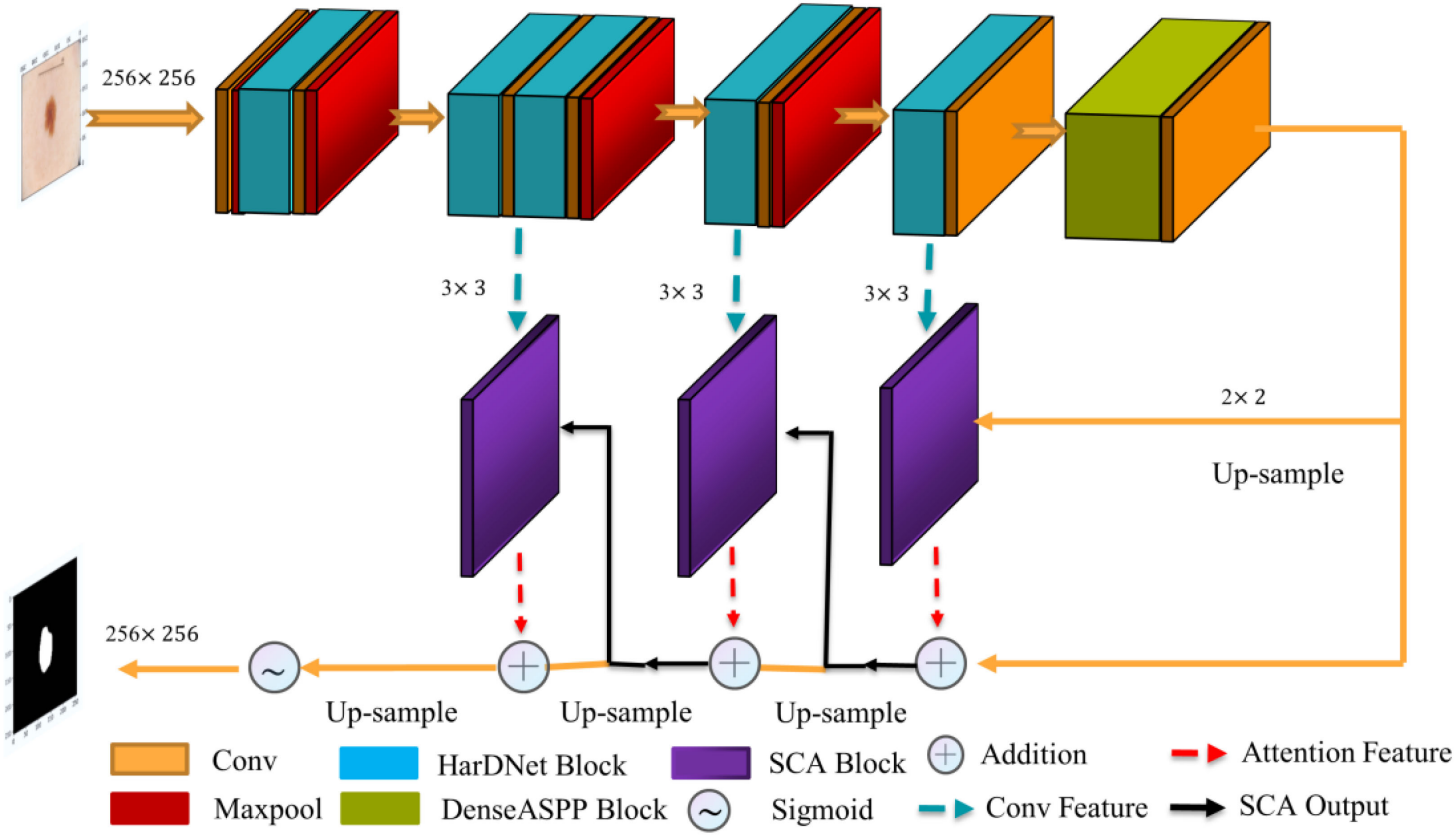
The proposed architecture.

The proposed polyp image segmentation network is based on the traditional U-Net network structure, including the encoder-decoder structure. Incorporating the HarDNet68 structure, the attention mechanism module, and the multi-scale null convolution module into the network, as shown in **Figure 1**. The input image is subjected to a 3×3 convolution operation, followed by five consecutive HarDNet integration blocks. HarDNet68 improves the global dense connection of the original DenseNet into a sparse connection with the convolution, BN, and ReLU activation functions to achieve repetition of batch normalization, reducing the number of parameters to obtain shorter inference speed while maintaining accuracy. The output of the encoder is used as the input of the multi-scale cavity convolution module, thus capturing more visual information of different scale features while capturing feature information of different scales. The decoder consists of three SCA modules, which obtains the spatial and attentional feature outputs on the channels by dot-product operations from the output of the previous stage and the corresponding edge outputs in HarDNet68. The Sigmoid function obtains the final segmentation result at the output of the decoder.

The segmentation network proposed in this paper forms an iterative interaction mechanism between the encoder and the decoder, which can effectively correct the conflicting regions such as boundaries in the prediction results thus improving the segmentation accuracy, as well as enhancing the inference speed and computational efficiency of the model.

### 2.1 U-net

**Figure 2:**
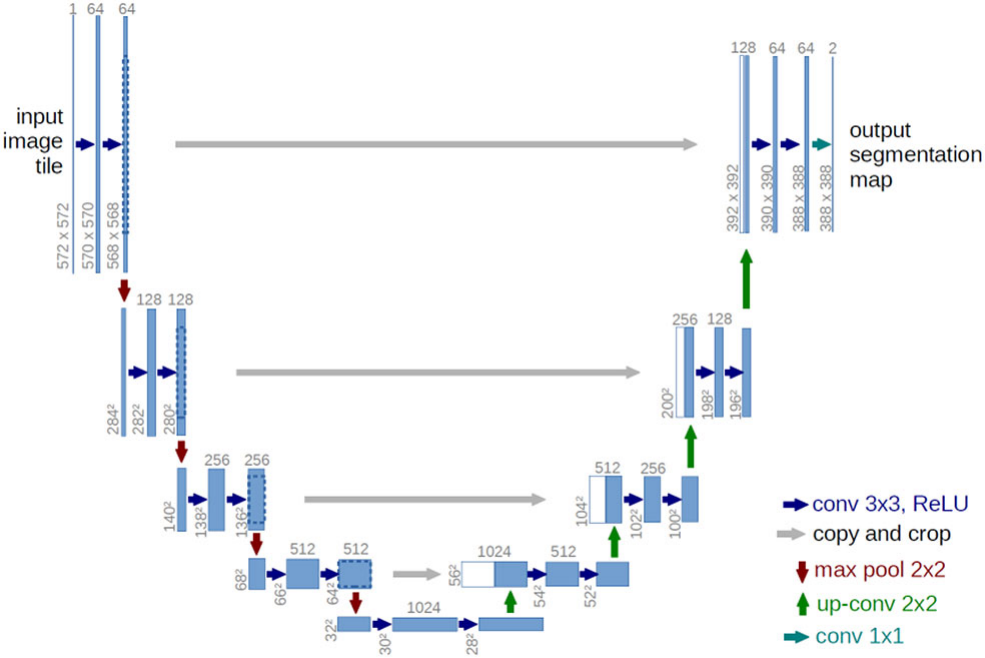
U-net architecture (29).

The U-net network structure (30) is all convolutional layers without fully connected layers, as shown in **Figure 2**. In the encoder part, the feature of the image is continuously extracted through the convolution layer, and the size of the feature image is also reduced. In the decoder part, the image is restored to the original size by de-convolution, and in the decoding part, the same size of the feature map in the encoding process is connected through cross-links, so that the image information features are lost as little as possible.

### 2.2 HardNet

**Figure 3:**
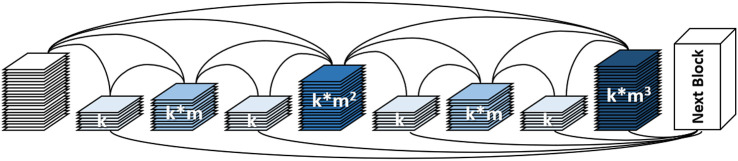
HarDNet network structure (27).

HarDNet structure, a harmonic densely connected network proposed by Chao et al. in 2019 (27), is optimized and improved based on the DenseNet network structure, optimization and improvement are made to improve the model running speed, as shown in **Figure 3**.

HarDNet uses a sparse connection, assuming that layer k is connected to layer , 2^n^ which can divide k integer, where n≥0, k–2^n^≥0. With this connection, if 2^n^ is processed, layer 1 to layer 2^n^–1 can be cleared from memory, reducing the amount of model parameter computation. At the same time, HarDNet balances the channel ratio between the input and output of key layer layers by increasing the number of channels of some key layers to reduce the amount of model memory access.

According to the above design ideas, six HarDNet structures with different parameter settings are proposed, and the HarDNet68 structure is used in this paper. **Table 1** shows the parameter settings of HarDNet68.

**Table 1:** HarDNet68 parameters.

m	Stride 2	Stride 4	Stride 8	Stride 16	Stride32
1.7	3×3,32, Stride=2	8(HDB), k=14,t=128	16(HDB), k=16,t=256	16(HDB), k=40,t=640	4(HDB), k=160,t=1024
	3×3,64		16(HDB), k=20,t=320		

Multiplier m is the low-dimensional compression factor, “3×3, 32” means 32 convolutional layers with 3×3, HDB is the number of HarDNet modules, k is the growth rate, and t is the number of output channels of 1 × 1 convolutional transition.

### 2.3 DenseASPP

In the deep learning network model, the size of the image is generally uniform by stretching or cropping, but this causes information loss, distortion, and many other problems. He et al. proposed the Spatia Pyramid Pooling (SPP) structure, which is to use multiple pooling layers of different scales for feature extraction and fusion into an n-dimensional vector input to a fully connected layer (31). The Google team proposed the Atrous Spatia Pyramid Pooling (ASPP) structure based on the characteristics of multi-scale information and extension convolution in the DeepLab (32) series of work. ASPP introduces the concept of void convolution on the basis of SPP and further improves the ability of image feature information extraction.

**Figure 4:**
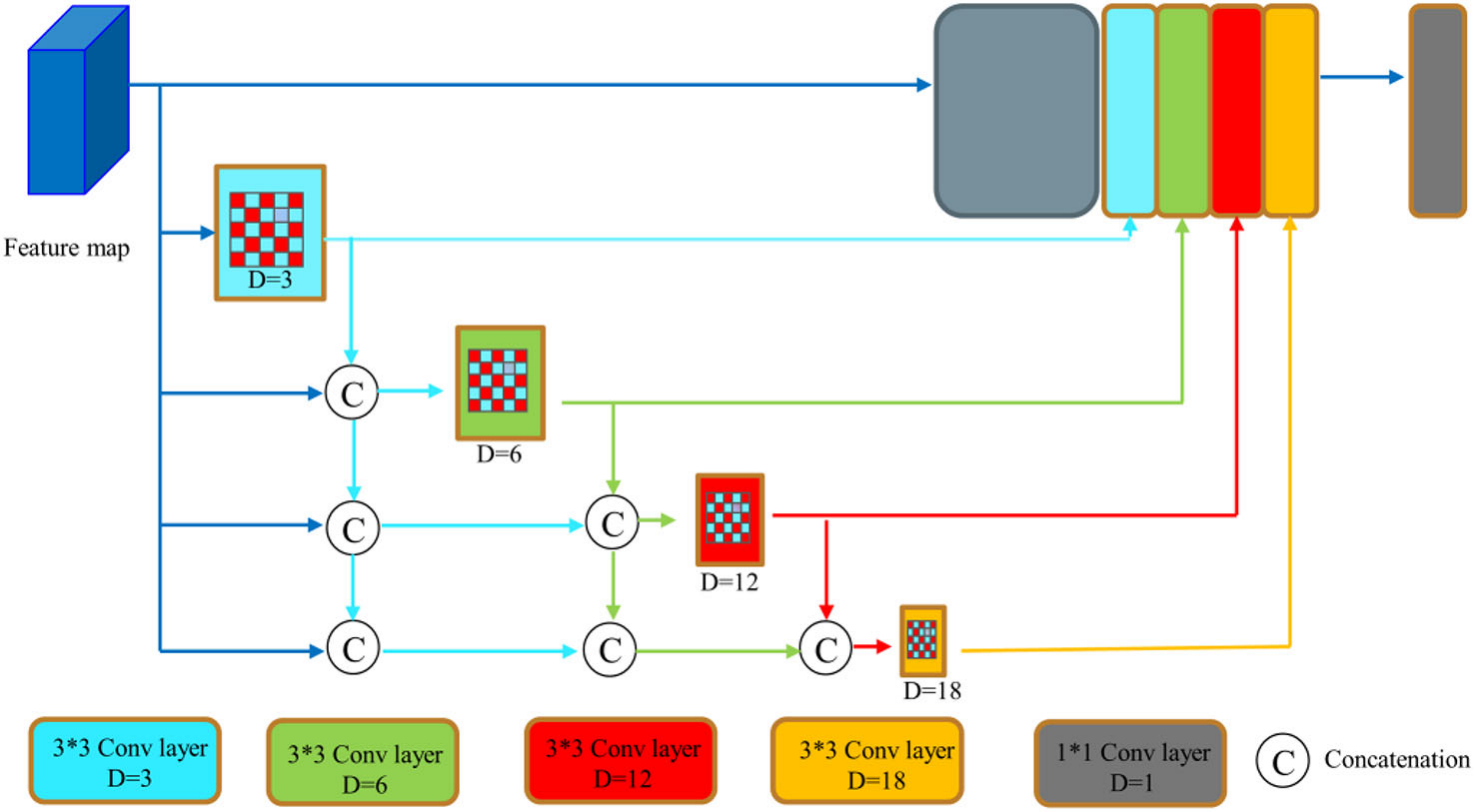
DenseASPP network structure (32).

However, the polyps have various shapes and textures, and the picture background is complicated, which easily causes poor segmentation effect in the process of polyp image segmentation. We introduces the DenseASPP module (33) to replace the ASPP module, which has the network structure shown in **Figure 4**. DenseASPP further increases the denseness of the null convolution and expands the range of the network model, improving the ability of the network model to extract image feature information without significantly increasing the model size. Since using a null convolution with too large an expansion rate can lead to convolutional degradation and cause degradation in feature extraction performance, only null convolutional layers with expansion rates of 3, 6, 12, and 18 are used in this paper.

### 2.4 Attention mechanism

When human beings observe things, their attention will focus on the areas of interest and ignore other areas. Through the attention mechanism, the input information is selectively distinguished, located, and analyzed. In the process of neural network learning, the attention mechanism will also be applied in the fields of image segmentation, target tracking, and behavior detection. We also can obtain the image feature information of different spaces and latitudes by giving different weight information to the input image, to improve the adaptability and effect of the neural network model (34, 35). How to build an attention mechanism model and integrate it into the mainstream neural network structure, so that the simple neural network can achieve complex and high-precision image segmentation tasks, is one of the problems that need to be solved.

#### 2.4.1 Spatial attention

After analyzing the input image, the neural network assigns more weights to the regions which are closely related to the segmentation task, which makes the target segmentation region more prominent. At the same time, the image region feature information which has nothing to do with the segmentation task is suppressed. After the maximum and average pooling operations, the two pooling results are stitched together to achieve the input image feature information fusion. Then, the convolution kernel of 1×1 is multiplied by the fused feature information, the sigmoid activation function is used for nonlinear transformation, and the spatial attention weight map is obtained. Finally, the input feature information of the image is multiplied by the spatial attention weight map to get the final output result, the formula is shown in Eq. (1) to Eq. (4).


(1) $${\text{x}}_{m}=Maxpool\left(x_{input}\right)$$

(2) $${\text{ x}}_{a}=Avgpool\left(x_{input}\right)$$

(3) $${\text{x}}_{graphs}=Sigmoid\left({\text{w}}_{1}*\left(cat\left[x_{m},x_{a}\right]\right)+b_{1}\right)$$

(4) $${\text{x}}_{outputs}={\text{x}}_{graphs}*x_{input}$$

x*_m_* and x*_a_*, are obtained using maximum pooling and average pooling operations on the feature map. *x_input_* represents the input feature maps. x*_graphs_* represents that the feature map obtained after nonlinear transformation of the fused image features with the sigmoid activation function.

x*_outputs_* represents the output of the input image multiplied with the spatial attention weight information.

#### 2.4.2 Channel attention

In the neural network, the corresponding channel represents the image feature information. Convolution kernels of different scales process the input image features to generate different image feature channel information. The feature information on each channel is different, and the importance of the global feature information of the whole image is also different. Through the analysis of the segmented image, we can assign weight information to each channel information, indicating the importance of the channel information to the global feature description. For the input image feature information, firstly, the dimensionality reduction operation is carried out by maximum pooling and average pooling, and the two feature infor            *x*_*m*_=*Maxpool*(*x*_*input*_) mation is input into a shared network structure for processing. The convolution kernel of 1×1 is multiplied by the fused feature information, the Sigmoid activation function is used for nonlinear transformation, and the channel attention weight map is obtained. Finally, the input feature information of the image is multiplied by the channel attention weight map to get the final output result, the formula is shown in Eq. (5) to Eq. (10).


(5) $$x_{m}=Maxpool\left(x_{input}\right)$$

(6) $$x_{a}=Avgpool\left(x_{input}\right)$$

(7) $${\text{x}}_{1}={\text{w}}_{\text{fc}3}*\left({\text{w}}_{\text{fc}2}*\left({\text{w}}_{\text{fc}1}{*\text{x}}_{\text{m}}+{\text{b}}_{\text{fc}1}\right)+{\text{b}}_{\text{fc}2}\right)+{\text{b}}_{\text{fc}3}$$

(8) $$x_{2}=w_{fc3}*\left(w_{fc2}*\left(w_{fc1}*x_{a}+b_{fc1}\right)+b_{fc2}\right)+b_{fc3}$$

(9) $$x_{graphc}=Sigmoid\left(w_{f}*\left(cat\left[x_{1},x_{2}\right]\right)+b_{f}\right)$$

(10) $$x_{outputc}=x_{graphc}*x_{input}$$

x*_m_* and x*_a_*, are obtained using maximum pooling and average pooling operations on the feature map. *x^input^* represents the input feature maps. x*_graphs_* represents that the feature map obtained after nonlinear transformation of the fused image features with the sigmoid activation function. *w*_*fc*1_∈*R* ^*C*/8×1×1^ ,  *w*_*fc*2_∈*R* ^*C*/8×1×1^ ,  *w*_*fc*3_∈*R* ^*C*×1×1^ .x*_outputs_* represents the output of the input image multiplied with the spatial attention weight information.

#### 2.4.3 Spatial channel attention

In the paper, the channel attention and spatial attention mechanism are combined and given different weights. Finally, the output image feature information processed by the SCA module is as Eq. (11):


(11) $${\text{x}}_{output}=cat\left[{\text{x}}_{outputs},{\text{x}}_{outputc}\right]$$

x*_outputs_* represents the output of the input image multiplied with the spatial attention weight information.

x*_outputc_* represents the output of the input image multiplied with the channel attention weight information.

Our proposed SCA module (36), with a general design idea similar to the architecture proposed by Fu et al. (37), integrates spatial and channel attention integration modules into an improved U-net network structure. The SCA module combines spatial and channel attention mechanisms to get comprehensive attention mechanism information. This module enhances the significant features of the up-sampling process by applying attention weights to high-dimensional and low-dimensional image feature information.

**Figure 5:**
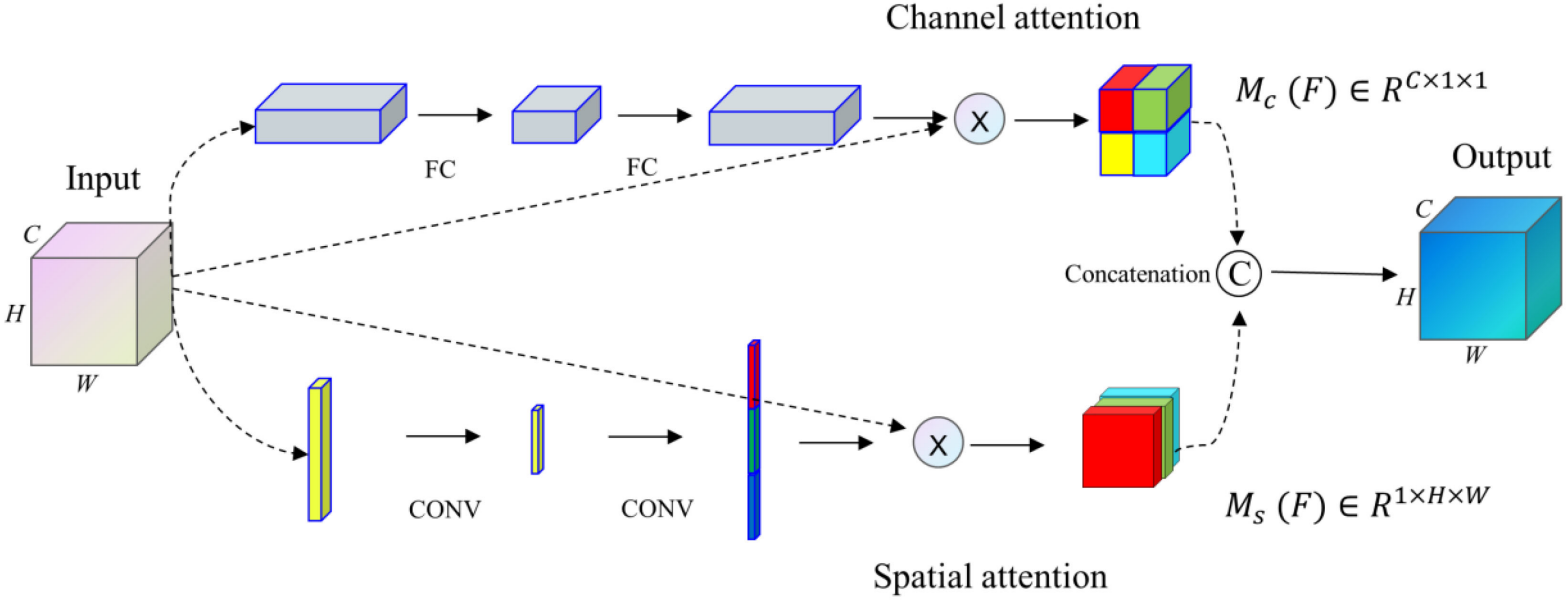
The SCA architecture.

The feature map x∈R^C×H×W^ as input, the attention weights  M_c_ (F)∈R^C×1×1^  and weights M_s_ (F)∈R^1×H×W^  in the channel and space are obtained respectively through the SCA module. Finally, the results of the two modules are operated by concatenation, as shown in **Figure 5**.

The SCA module proposed in the paper can address the feature information of medical images, highlight more of the key feature information of medical images, and suppress the interference of noise factors in medical images.

### 2.5 Loss function

The loss function is the function of a neural network to measure the degree of loss and error, and it is the index of a neural network to find the optimal weight parameters (38). Through the loss function, the difference between the segmentation result of the model and the actual result can be reflected. There are many kinds of loss functions, including single loss function and mixed loss function. The loss function adopted in this paper is the combined loss function of Dice loss (39) and Focal loss (40). This function can combine the advantages of the two functions to make the network better find the optimal parameters for optimization learning *ℒ*_Diceloss_ is the loss function of Dice loss. It is mainly used to measure the degree of loss of similarity between the segmented image predicted by the model and the real segmented image, and the value range is [0,1]. The calculation formula of the function is shown in Eq. (12). $\left|X\cap^{}\text{Y}\right|$ represents the number of intersections between a real segmented image and a model predicted image, |*X*| and |*Y*| represent the number of real segmented images and model predicted images respectively.


(12) $${ℒ}_{\text{Diceloss}}=1-\frac{2\left|X\cap^{}\text{Y}\right|}{\left|X\right|+\left|Y\right|}$$

*ℒ*_Focalloss_ is a loss function to deal with the unbalanced classification of samples. According to the difficulty of sample resolution, different weight coefficients α are added to the samples to reduce the adverse effects on training loss caused by the imbalance of sample classification. The calculation formula of the function is shown in Eq. (13).


(13) $${ℒ}_{\text{Focalloss}}=-\text{α}\times {\left(1-\text{p}\right)}^{\gamma }\times \text{log}\left(p\right)$$

p∈[0,1] is the model’s probability of predicting the positive sample.  α∈[0,1] is used to balance the uneven proportions of positive and negative samples themselves. is used to adjust the rate of weight reduction for simple samples. *ℒ*_Focalloss_ is a modification of the cross entropy loss function. The total loss function proposed in this paper is *ℒ*_Total_ , the formula is shown in Eq. (14).


(14) $${ℒ}_{\text{Total}}={ℒ}_{\text{Diceloss}}+{ℒ}_{\text{Focalloss}}$$

## 3 Experimental results and analysis

### 3.1 Datasets and preprocessing

To evaluate the model performance and verify the effectiveness of the algorithm, the model in this paper was subjected to relevant experiments on the Kvasir dataset (41), CVC-ClinicDB dataset (13), ETIS-Larib dataset (42), CVC-ColonDB (13) and CVC-300 (43) datasets.

We increase the number of training samples by the data augmentation method to improve the training effect of the model.

Firstly, the data of Kvasir and CVC-ClinicDB datasets are combined respectively, and then the training set, validation set and test set are divided according to the ratio of 80%, 10%, and 10%. CVC-ColonDB, ETIS-Larib, and CVC-300, are only used as the test set and do not participate in the dataset division. We expand the number of images in the training set by operations such as data enhancement, including center cropping, random rotation, Gaussian blurring etc. Among, center cropping size is (160,160), random rotation is 30 degrees, and Gaussian blurring kernel size is (3,3). A single image can be augmented into 20 different images, the original number of images in the Kvasir dataset is 800, and 16,000 after data enhancement; the original number of images in the CVC-ClinicDB dataset is 490, and 9,800 after data enhancement.

In the above datasets, polyps are highly variable in shape, size, structure, and orientation, and the boundaries between them and the background are very blurred and difficult to distinguish, which poses a great challenge to accurate polyp segmentation. The model resizes the resolution of colonoscopy images with different resolutions at the time of input and resizes all images uniformly to 256×256 size.

### 3.2 Training and evaluation metrics

In the experimental part, the settings of all models are kept the same. The hardware device parameters of the algorithm running environment in this paper are: the processor is Intel i5-12400F, the graphics card is NVIDIA GeForce RTX2080T, and the deep learning framework is PyTorch1.6 framework. The network training process uses small batch training iterations, the batch size is set to 4, and the total number of training rounds is 300. The training is terminated early when the validation set accuracy no longer gets better for 50 consecutive rounds. The weighted sum of Dice loss and Focal loss function is used as the loss function, and the early stop method is triggered. We use the Adam optimization algorithm, and the learning rate set to 1e^-4^.

X is the set of pixels of the polyp region in the predicted segmentation result, and Y is the set of pixels of the gold standard polyp region in the original polyp image. Segmentation Results (SR) is the set of pixels predicted by the model for polyp segmentation, Ground Truth (GT) is the set of pixels for actual polyp segmentation, TP is the pixels correctly segmented in the polyp segmentation results, TN is the pixels incorrectly segmented in the polyp segmentation results, FP is the background pixels incorrectly treated as polyp pixels in the polyp segmentation results, and FN is the polyp pixels incorrectly treated as background pixels in the segmentation results. n is the number of images in the test set.

Several evaluation metrics of segmentation are used in the experiments, and the specific definitions of these metrics are given below.

Dice Similarity Coefficient (DSC): Calculates the similarity between the predicted target region and the actual target region. In this paper, the sum of the similarity coefficients of all test results in the test set is averaged and denoted as mDice. The relevant formula for the similarity coefficient is calculated as follows:


(15) $$\text{mDice}=\frac{1}{\text{n}}\left(\sum_{\;}^{\;}\overset{\text{n}}{\underset{\text{i}=1}{\;}}|\frac{2|X\cap Y|}{\left|X\right|+\left|Y\right|}|\right)$$

Intersection over Union (IoU): Calculates the ratio of the intersection of the two sets of predicted and actual values to the concurrent set. In this paper, the sum of the Intersection-over-Union coefficients of all the test results in the test set is averaged and denoted as mIoU. The relevant formula for the Intersection-over-Union coefficient is calculated as follows:


(16) $$\text{mIou}=\frac{1}{\text{n}}\left(\sum_{\;}^{\;}\overset{\text{n}}{\underset{\text{i}=1}{\;}}|\frac{\text{X}\cap \text{Y}}{\left|X\right|+|Y|-\left|\text{X}\cap \text{Y}\right|}|\right)$$

Sensitivity (sens): which indicates the ratio of the number of pixels correctly segmented to the number of all pixels in the image segmentation result, is calculated by the relevant formula as follows:


(17) $$\text{mIou}=\frac{1}{\text{n}}\left(\sum_{\;}^{\;}\overset{\text{n}}{\underset{\text{i}=1}{\;}}|\frac{\text{X}\cap \text{Y}}{\left|X\right|+|Y|-\left|\text{X}\cap \text{Y}\right|}|\right)$$

Specificity (spe): which indicates the ratio of the number of pixels correctly identified as incorrectly segmented to the number of all incorrectly segmented pixels in the segmentation result, is calculated by the relevant formula as follows:


(18) $$\text{spe}=\frac{1}{\text{n}}\left(\sum_{\;}^{\;}\overset{\text{n}}{\underset{\text{i}=1}{\;}}\left|\frac{\text{TN}}{\text{TN}+\text{FP}}\right|\right)$$

The structural similarity measure Sm is used to assess the similarity between predicted and manually labeled real graphs (44).


(19) $$\text{Sm}=\frac{1}{\text{n}}\left(\sum_{\;}^{\;}\overset{\text{n}}{\underset{\text{i}=1}{\;}}\left|\text{α}\times {\text{S}}_{\text{O}}+\left(1-\text{α}\right)\times {\text{S}}_{\text{R}}\right|\right)$$

where α is taken as 0.5; S_R_ and S_O_ are calculated by the structural similarity metrics in the field of image quality evaluation (44) denoting region-oriented and object-oriented structural similarity, respectively.

Mean Absolute Error (MAE): compare the pixel-by-pixel absolute difference between the predicted value y and the actual value y.


(20) $$\text{MAE}=\frac{1}{\text{n}}\left(\sum_{\;}^{\;}\overset{\text{n}}{\underset{\text{i}=1}{\;}}\left|\text{GT}-\text{SR}\right|\right)$$

### 3.3 Analysis of experimental results

#### 3.3.1 Comparative analysis of different algorithm models

To verify the performance and segmentation effect of the algorithm, the segmentation results on the Kvasir and CVC-ClinicDB polyp image datasets were compared with those of Unet (29), UNet++ (16), SFA (18), PraNet (25), UACANet-L (45), and UACANet-S (45) networks, respectively. **Table 2** shows the final obtained methods of each of segmentation performance metrics, where the bolded values indicate the optimal metrics.

**Table 2:** Comparison of segmentation effects of different methods on Kvasir and CVC-ClinicDB datasets.

Datasets	Methods	mDice	mIou	sens	spe	Sm	MAE	Time
Kvasir	UNet (29)	0.818 ± 0.058	0.746 ± 0.069	0.887 ± 0.061	0.941 ± 0.057	0.858 ± 0.062	0.055 ± 0.003	~8.3h
	UNet++ (16)	0.821 ± 0.061	0.763 ± 0.073	0.900 ± 0.059	0.956 ± 0.072	0.862 ± 0.058	0.048 ± 0.002	~9.2h
	PraNet (25)	0.898 ± 0.072	0.840 ± 0.120	0.899 ± 0.056	0.969 ± 0.047	0.915 ± 0.082	0.030 ± 0.003	~8.5h
	SFA (18)	0.723 ± 0.124	0.611 ± 0.097	0.874 ± 0.106	0.932 ± 0.053	0.782 ± 0.069	0.075 ± 0.004	~13.1h
	UACANet-L (45)	0.912 ± 0.071	0.859 ± 0.083	0.907 ± 0.096	0.958 ± 0.084	0.917 ± 0.058	0.025 ± 0.002	~11.4h
	UACANet-S (45)	0.905 ± 0.069	0.852 ± 0.086	0.909 ± 0.078	0.959 ± 0.063	0.914 ± 0.083	0.026 ± 0.002	~9.7h
	Ours	**0.915 ± 0.085**	**0.862 ± 0.120**	**0.911 ± 0.086**	**0.968 ± 0.081**	**0.922 ± 0.074**	**0.024 ± 0.001**	~7.5h
CVC-ClinicDB	UNet (29)	0.823 ± 0.654	0.755 ± 0.073	0.886 ± 0.061	0.943 ± 0.059	0.889 ± 0.072	0.019 ± 0.003	~6.2h
	UNet++ (16)	0.794 ± 0.058	0.729 ± 0.075	0.893 ± 0.082	0.961 ± 0.079	0.873 ± 0.083	0.022 ± 0.002	~7.3h
	PraNet (25)	0.899 ± 0.103	0.849 ± 0.093	0.935 ± 0.062	0.974 ± 0.072	0.936 ± 0.063	0.009 ± 0.001	~6.5h
	SFA (18)	0.700 ± 0.120	0.607 ± 0.104	0.879 ± 0.093	0.937 ± 0.038	0.793 ± 0.105	0.042 ± 0.002	~11.1h
	UACANet-L (45)	0.926 ± 0.073	0.880 ± 0.071	**0.941 ± 0.052**	0.985 ± 0.013	0.943 ± 0.032	0.006 ± 0.003	~9.6h
	UACANet-S (45)	0.916 ± 0.580	0.870 ± 0.063	0.927 ± 0.061	**0.989 ± 0.011**	0.940 ± 0.043	0.008 ± 0.004	~7.3h
	Ours	**0.931 ± 0.046**	**0.892 ± 0.092**	0.933 ± 0.047	0.984 ± 0.092	**0.945 ± 0.051**	**0.005 ± 0.002**	~5.5h

From **Table 2**, our proposed algorithm achieved good results on all six evaluation metrics on the Kvasir dataset, mDice, mIou, sens, spe, Sm and MAE were 0.915, 0.862, 0.911, 0.968, 0.922 and 0.024, respectively. Compared with the SFA network structure, mDice, mIou, sens, spe and Sm improve by 0.192, 0.251, 0.037, 0.036 and 0.140, respectively, and MAE decreases by 0.051. On the CVC-ClinicDB dataset, mDice, mIou, sens, spe, Sm and MAE were 0.931, 0.892, 0.933, 0.984, 0.945, and 0.005, respectively. Compared to the SFA network structure, mDice, mIou, sens, spe, and Sm improved by 0.231, 0.285, 0.054, 0.047, and 0.152, respectively, and MAE decreased by 0.037. The UACANet-L and UACANet-S network structures improve the metrics sens and spe metrics by 0.008 and 0.005, respectively. On the two datasets, the running time of the model in this paper is the least.

#### 3.3.2 Generalization performance

To verify the generalization performance of the algorithm, the unseen datasets (CVC-ColonDB, CVC-300, ETIS-Larib) are used to test the generalization ability of the model (the model training data are only from Kvasir and CVC-ClinicDB). From **Table 3**, we can found that the generalization ability of UNet, UNet++, and SFA is poor on the three datasets, especially the evaluation metrics of SFA decreases sharply, while the algorithm proposed in this paper shows superior levels of all indexes on the three test sets and achieves good results. Where the bolded values indicate the optimal metrics.

**Table 3:** Comparison results of different methods on an unseen dataset.

Datasets	Methods	mDice	mIou	sens	spe	Sm	MAE
CVC-ColonDB	UNet (29)	0.512	0.444	0.754	0.853	0.712	0.061
	UNet++ (16)	0.483	0.410	0.735	0.846	0.691	0.064
	PraNet (25)	0.709	0.640	0.821	0.914	0.819	0.045
	SFA (18)	0.469	0.347	0.716	0.842	0.634	0.094
	UACANet-L (45)	0.751	0.678	0.837	0.927	0.835	0.039
	UACANet-S (45)	0.783	0.704	0.841	0.936	0.848	0.034
	Ours	**0.805**	**0.722**	**0.849**	**0.938**	**0.858**	**0.031**
CVC-300	UNet (29)	0.710	0.627	0.897	0.923	0.843	0.022
	UNet++ (16)	0.707	0.624	0.915	0.931	0.839	0.018
	PraNet (25)	0.871	0.797	0.938	0.966	0.925	0.010
	SFA (18)	0.467	0.329	0.887	0.925	0.640	0.065
	UACANet-L (45)	0.910	0.849	0.928	0.970	0.937	0.005
	UACANet-S (45)	0.902	0.837	0.931	0.975	0.934	0.006
	Ours	**0.923**	**0.857**	**0.945**	**0.981**	**0.946**	**0.003**
ETIS-Larib	UNet (29)	0.398	0.335	0.673	0.782	0.684	0.036
	UNet++ (16)	0.401	0.344	0.687	0.779	0.683	0.035
	PraNet (25)	0.628	0.567	0.765	0.807	0.794	0.031
	SFA (18)	0.297	0.217	0.524	0.723	0.557	0.109
	UACANet-L (45)	0.766	0.689	0.797	0.827	0.859	0.012
	UACANet-S (45)	0.694	0.615	0.801	0.789	0.815	0.023
	Ours	**0.774**	**0.691**	**0.812**	**0.834**	**0.864**	**0.009**

#### 3.3.3 Results visualization analysis

**Figure 6:**
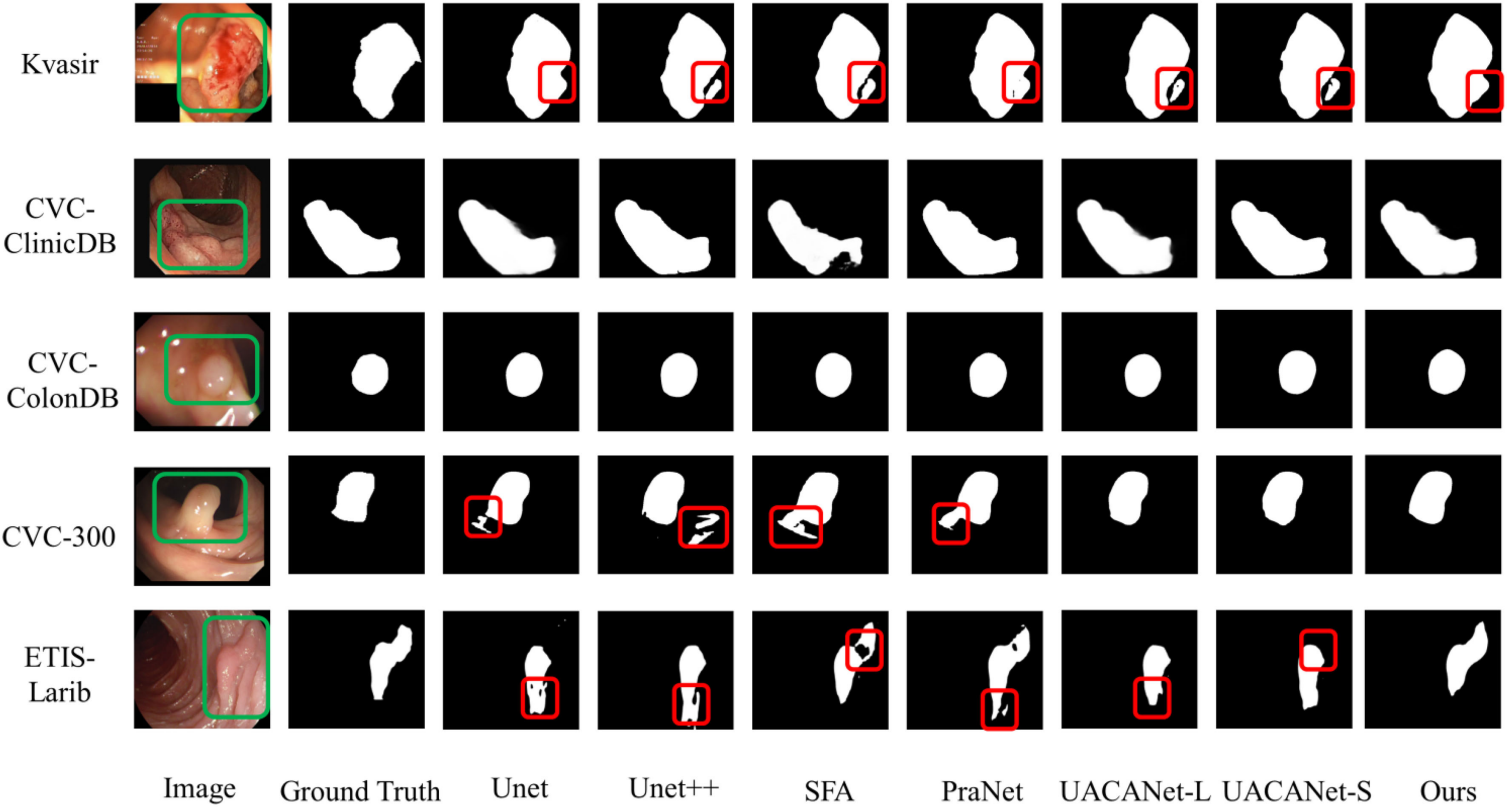
Polyp segmentation results.

The model can clearly distinguish polyps from other tissues in the polyp segmentation task, keep the polyp edge segmentation intact while reducing the miss segmentation inside the polyp, and the segmentation results are shown in **Figure 6**, and the red areas in the figure are the pixel points missed segmentation by the network. Some of them are segmentation networks that incorrectly mark background pixels as polyps; some of them are segmentation networks that incorrectly mark polyps pixels as background pixels.

From **Figure 6**, we can find that the segmentation model proposed in this paper can achieve better segmentation results. However, for images with smooth polyp edges that are easy to distinguish, such as the third row in **Figure 6**, all models are able to segment accurately. When the edges of the lesion region are similar to the background, all segmentation models have certain challenges and are prone to multi-segmentation or omission of pixel points. From the segmentation results in the first and second rows of **Figure 6**, it is found that our models have poor segmentation results; however, for some polyps images with complex backgrounds, our models can basically distinguish the lesion regions with blurred borders completely, as shown in the fourth and fifth rows of **Figure 6**.

From the visualized results, we can conclude that our proposed model can generally overcome the problem of polyps with similar colors and backgrounds well, and detect polyps with different shapes and sizes and colors of tissues, and the delineated areas and boundaries are more clear and accurate.

## 4 Discussion

We propose a network model for polyp image segmentation based on deep neural network techniques by combining HarDNet and a multiscale coding module of attention mechanism and perform comparison and generalization experiments on five polyp image datasets. The **Table 2** shows the segmentation results of UNet, UNet++, SFA, PraNet, UACANet-L, and UACANet-L networks on the Kvasir and CVCClinicDB polyp image datasets. **Table 3** shows the networks such as UNet, UNet++, SFA, PraNet, UACANet-L, and UACANet-L tested on the Kvasir and CVCClinicDB network models on the CVC-ColonDB, CVC-300, and ETIS-Larib datasets to verify the generalization performance of the models. **Figure 6** shows the segmentation results of each model on the Kvasir, CVC-ClinicDB, CVC-ColonDB, CVC-300, and ETIS-Larib polyp image datasets, respectively.

For the data analysis in **Tables 2**, **3**, and **Figure 6** above, we found that the polyp image segmentation model based on deep learning proposed in this paper can well overcome the problem of similar a color of polyps and backgrounds, detect polyp tissues with different shapes and sizes and colors, and achieve excellent results with clearer and more accurate delineation of regions and boundaries.

Through the analysis of the above results, we know that the U-Net network causes the semantic gap phenomenon due to the information difference between the output features in the encoding and decoding stages, which affects the segmentation results. Our proposed polyp segmentation network is able to capture the path of contextual information and effectively reduce the semantic discrepancy, which well overcomes the diversity of polyp shape, size, color and texture as well as the unclear boundary between the polyp and its surrounding mucosa to achieve more accurate segmentation results.

Our proposed polyp image segmentation network has achieved a certain degree of improvement, but the model still has room for further enhancement.

First, to improve the training speed of the network, we used parameters and weights based on ImageNet pre-training, but there are huge differences in features, textures, and other information between ordinary images and polyp images, which impose certain limitations on the effectiveness of medical image segmentation. Secondly, our network has not been attempted to be validated on a 3D medical image segmentation dataset. Finally, the size of polyp images in the polyp image dataset studied in this paper varies greatly, but we set a uniform size of 256×256 during image preprocessing and did not validate the effect of image size on model segmentation accuracy. In addition, we just merged the Kvasir and CVC-ClinicDB datasets and could not guarantee the independence between the subsets. We will further investigate these issues in our future work.

## 5 Conclusions

In the paper, a multi-scale coded colon polyp image segmentation network combining HarDNet and attention mechanism was proposed for automatic polyp segmentation of colonoscopy images, reducing the effects of orientation, shape, texture, and size on the results. The proposed segmentation network was evaluated on five polyp image datasets, just as the Kvasir, CVC-ClinicDB, CVC-ColonDB, CVC-300, and ETIS-Larib, and analyzed and compared with other existing representative methods. Through comparative analysis with other models, we can found that the accuracy of the segmentation algorithm proposed in this paper is better than other methods, and for images with very low contrast between polyps and surrounding mucosa. Through experimental comparison and analysis, the segmentation algorithm proposed has better accuracy than other methods. It can accurately segment the boundary of polyps even for images with very low contrast between polyps and surrounding mucosa. There are no image artifacts outside the boundary with good image coherence. The polyp segmentation network proposed has excellent performance and good generalization ability, which can assist physicians in the diagnosis of colorectal polyps and reduce the leakage and misdiagnosis in clinical time, and is of reference for the processing and analysis of colorectal polyp images.

## Data availability statement

The original contributions presented in the study are included in the article/supplementary material. Further inquiries can be directed to the corresponding author.

## Author contributions

TS did the conceptualisation, analysis and writing. XL provided conceptualisation, aided analysis and revised writing. All authors contributed to the article and approved the submitted version.

## References

[1] Sung H Ferlay J Siegel RL Laversanne M Soerjomataram I Jemal A. Global cancer statistics 2020: GLOBOCAN estimates of incidence and mortality worldwide for 36 cancers in 185 countries. Ca: Cancer J Clin (2021) 171:209–49. doi: 10.3322/caac.21660 33538338

[2] Lieberman DA Rex DK Winawer SJ Giardiello FM Johnson DA Levin TR. Guidelines for colonoscopy surveillance after screening and polypectomy: A consensus update by the US multi-society task force on colorectal cancer. Gastroenterology (2012) 143(3):844–57. doi: 10.1053/j.gastro.2012.06.001 22763141

[3] Corley DA Jensen CD Marks AR Zhao WK Lee JK Doubeni CA. Adenoma detection rate and risk of colorectal cancer and death. New Engl J Med (2014) 370:1298–306. doi: 10.1056/NEJMoa1309086 PMC4036494 24693890

[4] Jia X Xing X Yuan Y Xing L Meng MQ. (2019). Wireless capsule endoscopy: A new tool for cancer screening in the colon with deep-learning-based polyp recognition. Proceedings of the IEEE,. United States, IEEE,.Vol. 108. pp. 178–197. doi: 10.1109/JPROC.2019.2950506

[5] Saul C Canali C Teixeira CR Parada AA Prolla JC da Silva VD. Digital morphometric characterization of mucosal surface lesion patterns under magnification colonoscopy. Analytical Quantitative Cytology Histol (2009) 31:375–9. 20698353

[6] Bashar MK Kitasaka T Suenaga Y Mekada Y Mori K. Automatic detection of informative frames from wireless capsule endoscopy images. Med Image Anal (2010) 14:449–70. doi: 10.1016/j.media.2009.12.001 20137998

[7] Segui S Drozdzal M Vilarino F Malagelada C Azpiroz F Radeva P. Categorization and segmentation of intestinal content frames for wireless capsule endoscopy. IEEE Trans Inf Technol Biomedicine (2012) 16:1341–52. doi: 10.1109/TITB.2012.2221472 24218705

[8] Turcza P Duplaga. M. Hardware-efficient low-power image processing system for wireless capsule endoscopy. IEEE J Biomed Health Inf (2013) 13:1046–56. doi: 10.1109/JBHI.2013.2266101 24240723

[9] Hassan AR Haque MA. Computer-aided gastrointestinal hemorrhage detection in wireless capsule endoscopy videos. Comput Methods Programs Biomedicine (2015) 122:341–53. doi: 10.1016/j.cmpb.2015.09.005 26390947

[10] Qiao P Liu H Yan X Jia Z Pi X. A smart capsule system for automated detection of intestinal bleeding using hsl color recognition. PloS One (2016) 11:14. doi: 10.1371/journal.pone.0166488 PMC5130220 27902728

[11] Mamonov AV Figueiredo IN Figueiredo PN Tsai YH. Automated polyp detection in colon capsule endoscopy. IEEE Trans Med Imaging (2014) 33:1488–502. doi: 10.1109/TMI.2014.2314959 24710829

[12] Tajbakhsh N Gurudu S Liang J. Automated polyp detection in colonoscopy videos using shape and context information. IEEE Trans Med Imaging (2015) 35:630–44. doi: 10.1109/TMI.2015.2487997 26462083

[13] Bernal J Sánchez FJ Fernández-Esparrach G Gil D Rodríguez C Vilariño F. WM-DOVA maps for accurate polyp highlighting in colonoscopy: Validation vs. saliency maps from physicians. Computerized Med Imaging Graphics (2015) 43:99–111. doi: 10.1016/j.compmedimag.2015.02.007 25863519

[14] Brandao P Mazomenos E Ciuti G Caliò R Bianchi F Menciassi A. Fully convolutional neural networks for polyp segmentation in colonoscopy. In: Medical imaging 2017:Computer-aided diagnosis. Orlando, Florida, United States: SPIE (2017). p. 101–7. doi: 10.1117/12.2254361

[15] Wang L Qian Y Hu Y. IDDF2018-ABS-0259 segmentation of intestinal polyps via a deep learning algorithm. Int Digestive Dis Forum (IDDF) (2018), . 83–84. doi: 10.1136/gutjnl-2018-IDDFabstracts.180

[16] Zhou Z Siddiquee MMR Tajbakhsh N Liang JM. UNet++: A nested U-net architecture for medical image segmentation. In: Deep learning in medical image analysis and multimodal learning for clinical decision support. Granada, Spain: Springer. pp.3–11. doi: 10.1007/978-3-030-00889-5_1 PMC7329239 32613207

[17] Jha D Smedsrud PH Riegler MA Johansen D De Lange T Halvorsen P. (2019). Resunet++: An advanced architecture for medical image segmentation, in: 2019 IEEE International Symposium on Multimedia (ISM). San Diego, USA: IEEE. pp. 225–2255. doi: 10.1109/ISM46123.2019.00049

[18] Fang Y Chen C Yuan Y Tong KY. (2019). Selective feature aggregation network with area-boundary constraints for polyp segmentation, in: International Conference on Medical Image Computing and Computer-Assisted Intervention. Shenzhen, China: Springer. pp. 302–10. doi: 10.1007/978-3-030-32239-7_34

[19] Jha D Riegler MA Johansen D Halvorsen P Johansen HD. (2020). Doubleu-net: A deep convolutional neural network for medical image segmentation, in: 2020 IEEE 33rd International symposium on computer based medical systems (CBMS). Rochester, MN, USA: IEEE. pp. 558–64. doi: 10.1109/CBMS49503.2020.00111

[20] Nadimi ES Buijs MM Herp J Kroijer R Kobaek-Larsen M Nielsen E. Application of deep learning for autonomous detection and localization of colorectal polyps in wireless colon capsule endoscopy. Comput Electrical Eng (2020) 81:1–16. doi: 10.1016/j.compeleceng.2019.106531

[21] Owais M Arsalan M Mahmood T Kang JK Park KR. Automated diagnosis of various gastrointestinal lesions using a deep learning-based classification and retrieval framework with a large endoscopic database: Model development and validation. J Med Internet Res (2020) 22:1–21. doi: 10.2196/18563 PMC7728528 33242010

[22] Yamada A Niikura R Otani K Aoki T Koike K. Automatic detection of colorectal neoplasia in wireless colon capsule endoscopic images using a deep convolutional neural network. Endoscopy (2020) 17:1–19. doi: 10.1055/a-1266-1066 32947623

[23] Lee SA Cho HC. A novel approach for increased convolutional neural network performance in gastric-cancer classification using endoscopic images. IEEE Access (2021) 9:51847–54. doi: 10.1109/ACCESS.2021.3069747

[24] Lai LL Blakely A Invernizzi M Lin J Kidambi T Melstrom KA. Separation of color channels from conventional colonoscopy images improves deep neural network detection of polyps. J Biomed Optics (2021) 26:1–18. doi: 10.1117/1.JBO.26.1.015001 PMC7805485 33442965

[25] Fan DP Ji GP Zhou T Chen G Fu H Shen J. (2020). Pranet: Parallel reverse attention network for polyp segmentation, in: International conference on medical image computing and computer-assisted intervention. Lima, Peru: Springer. pp. 263–73. doi: 10.1007/978-3-030-59725-2_26

[26] Zhao X Zhang L Lu H. (2021). Automatic polyp segmentation via multi-scale subtraction network, in: International Conference on Medical Image Computing and Computer-Assisted Intervention. Strasbourg, France: Springer. pp. 120–30. doi: 10.1007/978-3-030-87193-2_12

[27] Chao P Kao CY Ruan YS Huang CH Lin YL. (2019). Hardnet: A low memory traffic network, in: Proceedings of the IEEE/CVF international conference on computer vision. Seoul, Korea: IEEE. pp. 3552–61. doi: 10.1109/ICCV.2019.00365

[28] Huang CH Wu HY Lin YL. Hardnet-mseg: A simple encoder-decoder polyp segmentation neural network that achieves over 0.9 mean dice and 86 fps. arXiv preprint ar (2021) Xiv:2101.07172. doi: 10.48550/arXiv.2101.07172

[29] Srivastava A Jha D Chanda S Pal U Johansen HD Johansen D. Msrf-net: A multi-scale residual fusion network for biomedical image segmentation. IEEE J Biomed Health Inf (2021) 26(5):2252–63. doi: 10.1109/JBHI.2021.3138024 34941539

[30] Ronneberger O Fischer P Brox T. (2015). U-net: Convolutional networks for biomedical image segmentation, in: International Conference on Medical image computing and computer-assisted intervention. Munich, Germany: Springer. pp. 234–41. doi: 10.1007/978-3-319-24574-4_28

[31] He K Zhang X Ren S Sun J. Spatial pyramid pooling in deep convolutional networks for visual recognition. IEEE Trans Pattern Anal Mach Intell (2014) 37:1904–16. doi: 10.1109/TPAMI.2015.2389824 26353135

[32] Chen LC Zhu Y Papandreou G Schroff F Adam H. Encoder-decoder with atrous separable convolution for semantic image segmentation. Comput Vision-ECCV 2018 (2018), 801–18. doi: 10.1007/978-3-030-01234-2_49

[33] Yang M Yu K Zhang C Li Z Yang K. (2018). DenseASPP for semantic segmentation in street scenes, in: 2018 IEEE/CVF Conference on ComputerVision and Pattern Recognition. Salt Lake City, UT, USA: IEEE. pp. 3684–92. doi: 10.1109/CVPR.2018.00388

[34] Huang QH Huang YH Luo YZ Yuan FN Li XL. Segmentation of breast ultrasound image with semantic classification of superpixels. Med Image Anal (2020) 61:101657. doi: 10.1016/j.media.2020.101657 32032899

[35] Huang QH Miao ZJ Zhou SC Chang C Li XL. Dense prediction and local fusion of superpixels: A framework for breast anatomy segmentation in ultrasound image with scarce data. IEEE Trans Instrumentation Measurement (2021) 70:1–8. doi: 10.1109/TIM.2021.3088421

[36] Shen TP Xu HQ. Facial expression recognition based on multi-channel attention residual network. CMES-Computer Modeling Eng Sci (2023) 135:539–60. doi: 10.32604/cmes.2022.022312

[37] Fu J Liu J Tian H Li Y Bao Y Fang Z. (2019). Dual attention network for scene segmentation, in: Proceedings of the IEEE/CVF conference on computer vision and pattern recognition. Long Beach, Canada: IEEE. pp. 3146–54. doi: 10.1109/CVPR.2019.00326

[38] Jun M Chen JN Ng M Huang R Li Y Li C. Loss odyssey in medical image segmentation. Med Image Anal (2021) 71:102035. doi: 10.1016/j.media.2021.102035 33813286

[39] Wang R Lei T Cui R Zhang B Meng H Nandi AK. Medical image segmentation using deep learning: A survey. IET Image Process (2022) 162:1243–67. doi: 10.1049/ipr2.12419

[40] Lin TY Goyal P Girshick R He K Dollár P. (2017). Focal loss for dense object detection, in: Proceedings of the IEEE international conference on computer vision. Venice, Italy: IEEE. pp. 2980–8. doi: 10.1109/TPAMI.2018.2858826

[41] Jha D Smedsrud PH Riegler MA Halvorsen P Lange TD Johansen D. (2020). Kvasir-seg : A segmented polyp dataset, in: International Conference on Multimedia Modeling, Daejeon, South Korea. pp. 451–62.

[42] Silva J Histace A Romain O Dray X Granado B. Toward embedded detection of polyps in WCE images for early diagnosis of colorectal cancer. Int J Comput Assisted Radiol Surg (2014) 9:283–93. doi: 10.1007/s11548-013-0926-3 24037504

[43] Vázquez D Bernal J Sánchez FJ Fernández-Esparrach G López AM Romero A. A benchmark for endoluminal scene segmentation of colonoscopy images. J healthcare Eng (2017) 2017:4037190. doi: 10.1155/2017/4037190 PMC5549472 29065595

[44] Fan DP Cheng MM Liu Y Li T Borji A. (2017). Structure-measure: A new way to evaluate foreground maps, in: Proceedings of 2017 IEEE International Conference on Computer Vision, Venice, Italy: IEEE. pp. 4558–67. doi: 10.1109/ICCV.2017.487

[45] Kim T Lee H Kim D. (2021). UACANet: Uncertainty augmented context attention for polyp segmentation, in: Proceedings of the 29th ACM International Conference on Multimedia, New York, USA: ACM. pp. 2167–75. doi: 10.1145/3474085.3475375

